# Amine Analysis Using AlexaFluor 488 Succinimidyl Ester and Capillary Electrophoresis with Laser-Induced Fluorescence

**DOI:** 10.1155/2015/368362

**Published:** 2015-05-19

**Authors:** Christian G. Kendall, Amanda M. Stockton, Stephen Leicht, Heather McCaig, Shirley Chung, Valerie Scott, Fang Zhong, Ying Lin

**Affiliations:** ^1^Jet Propulsion Laboratory, California Institute of Technology, Pasadena, CA 91109, USA; ^2^Weill Cornell Graduate School of Medical Science, New York, NY 10065, USA; ^3^Georgia Institute of Technology, Atlanta, GA 30332, USA; ^4^University of California, Los Angeles, CA 90095, USA

## Abstract

Fluorescent probes enable detection of otherwise nonfluorescent species via highly sensitive laser-induced fluorescence. Organic amines are predominantly nonfluorescent and are of analytical interest in agricultural and food science, biomedical applications, and biowarfare detection. Alexa Fluor 488 N-hydroxysuccinimidyl ester (AF488 NHS-ester) is an amine-specific fluorescent probe. Here, we demonstrate low limit of detection of long-chain (C_9_ to C_18_) primary amines and optimize AF488 derivatization of long-chain primary amines. The reaction was found to be equally efficient in all solvents studied (dimethylsulfoxide, ethanol, and N,N-dimethylformamide). While an organic base (N,N-diisopropylethylamine) is required to achieve efficient reaction between AF488 NHS-ester and organic amines with longer hydrophobic chains, high concentrations (>5 mM) result in increased levels of ethylamine and propylamine in the blank. Optimal incubation times were found to be >12 hrs at room temperature. We present an initial capillary electrophoresis separation for analysis using a simple micellar electrokinetic chromatography (MEKC) buffer consisting of 12 mM sodium dodecylsulfate (SDS) and 5 mM carbonate, pH 10. Limits of detection using the optimized labeling conditions and these separation conditions were 5–17 nM. The method presented here represents a novel addition to the arsenal of fluorescent probes available for highly sensitive analysis of small organic molecules.

## 1. Introduction

Quantitative compositional analysis of specific primary amines is applied in food science and agriculture to characterize samples for quality control. Primary amines in soil samples provide information about the available sources of organic and bioorganic N in an ecosystem [1], a boon for management of agriculture, and a central aspect to researching systems that involve N or the nitrogen fixation cycle. Primary amines and amino acids are also used to indicate reactions in food processing and to directly indicate nutritional value and quality of products [2–5]. A large majority of known bioactive molecules and neurotransmitters are primary amines, amino acids, or low molecular weight metabolites of these species [6], so primary amine analysis is of continually increasing interest for metabolomics, pharmaceuticals, and detection of hazardous agents in biowarfare.* In situ* analysis of primary amines is additionally of great interest for investigating planetary chemistry [7, 8] as well as the synthesis and origin of prebiotic amino acids [9].

For compositional amine analysis, a separation method must typically be applied to resolve specific amines within a sample. Many applications require field-deployable amine compositional analyses, such as clinical devices, detection of harmful biological agents, and* in situ *astrobiology and planetary science experiments. Capillary electrophoresis (CE), when used in conjunction with laser-induced fluorescence (LIF), provides a fast and easily miniaturizable technique. CE-LIF also provides the opportunity to incorporate the separation and detection steps in line with extraction instruments and microfluidics devices. This is an attractive feature for lab-on-a-chip approaches and a necessary step for developing field-deployable detection instruments for the clinic, biowarfare, and* in situ* astrobiology experiments. In fact, CE is already a targeted implementation of lab-on-a-chip analysis for bioterrorism defense [10], *μ*CE-LIF has been fully automated and miniaturized towards future* in situ *Martian and other planetary missions [7, 11], and CE-LIF's direct compatibility with dialysates and biological fluids has been applied to clinical samples and* in vivo* biomedical research [2, 12, 13]. However, the latter two applications often require detection of amines with varied solubility in aqueous media. Micellar electrokinetic chromatography (MEKC) enables separation and therefore analysis of hydrophobic longer-chain amines, while preserving analytic capability of shorter and more hydrophilic amines. MEKC-LIF, only differing from CE-LIF by addition of a surfactant, presents the opportunity to extend implementation of these established approaches to field-deployable instrument development to detect a broader spectrum of targets in complex samples.

Fluorescence detection of primary amines provides a quick and potentially highly sensitive, quantitative analysis. Particularly, fluorescence detection of amines by CE-LIF with excitation at 488 nm has demonstrated limits of detection (LOD) from *μ*M to nM [3] depending on the target amine, fluorescence probe, and optimization conditions. The speed of fluorescence detection ensures that the rate-limiting step for analysis is upstream in sample collection, preparation, or separation. However, to use this method, amines without autofluorescent properties must be chemically derivatized with a fluorescent probe. The commercially available dye, AlexaFluor 488 (AF488), is optimally excited with a 488 nm laser line (extinction coefficient of 73,000 cm^−1^ M^−1^ at 494 nm) and has an emission maximum at 525 nm, making it easy to apply with standard light sources, instrumental settings, and filters. Low AF488 self-quenching enables highly sensitive analysis, and the N-hydroxysuccinimidyl ester (NHS-ester) functionality makes it highly amine-specific. In aqueous and biological samples, the spontaneous derivatization reaction of AF488 NHS-ester circumvents the need for additional reagents while the high amine specificity provides advantages over other amine-reactive dyes such as isothiocyanates, which also react with sulfhydryl groups, and dyes which react directly with amines and change their chemical structure and fluorescent properties upon derivatization (e.g., 4-chloro-7-nitro-1,2,3-benzoxadiazole). The derivatization through amine esterification, promoted by the NHS leaving group, allows for modular selection of other fluorophores to optimize fluorescent properties for varied equipment. AF488 is a water soluble probe that is pH insensitive over a range that extends from below 4 to above 10, making it suitable for a range of applications. The dye is negatively charged overall, facilitating separation of tagged analytes by electromigration.

Here, we describe a novel method for amine analysis using MEKC-LIF in conjunction with a labeling protocol employing AF488 NHS-ester. We prepared and analyzed samples of nonylamine, hexadecylamine, and octadecyl amine to test the applicability of this method to aliphatic amines with reduced solubility in aqueous media and no detectable autofluorescence. Samples were separated and detected using a commercial Beckman Coulter P/ACE MDQ system with 488 nm LIF detection. Labeling conditions were optimized, including organic solvent, the concentration of the base diisopropylethylamine (DIEA), and the incubation time. Suitable separation characteristics were found using MEKC with sodium dodecyl sulfate as the surfactant, and the resulting analytical technique was characterized.

## 2. Materials and Methods

### 2.1. Materials

All chemicals were of analytical reagent grade and were used as received. AlexaFluor 488 succinimidyl ester (AF488 NHS-ester) was purchased from Invitrogen Corporation (Carlsbad, CA), diluted to 20 mM in N,N-dimethylformamide (DMF, Sigma-Aldrich, St. Louis, MO), and stored at −20°C. Sodium carbonate (NaCO_3_, Sigma-Aldrich) was used to prepare 50 mM aqueous solutions with 18 MΩ·cm water. The pH was adjusted using 1 M NaOH (Sigma-Aldrich) and measured using a glass electrode and a digital pH meter (Orion 290A, Thermo; Waltham, MA). Sodium dodecyl sulfate (SDS) was acquired from Sigma Aldrich and used to prepare a 100 mM stock in 18 MΩ·cm water. Amines for standard solutions, including nonylamine (C9-NH_2_), dodecylamine (C12-NH_2_), hexadecylamine (C16-NH_2_), and octadecylamine (C18-NH_2_), were purchased in pure form from Sigma Aldrich and used to prepare 10 mM solutions in ethanol (Sigma-Aldrich). N,N-diisopropylethylamine (DIEA, Sigma-Aldrich) was diluted to 10 mM in ethanol, DMF, and dimethylsulfoxide (DMSO, Sigma-Aldrich). The stock solutions were combined as needed to result in the solutions used.

### 2.2. Labeling Reactions

Labeling reactions were conducted by combining the appropriate volumes of 20 mM AF488, 10 mM DIEA, amine, and solvent. Reactions were incubated in the dark overnight (16–24 hrs) unless otherwise indicated. After incubation, reactions were diluted into the separation buffer at a 5 : 100 ratio unless otherwise indicated.

### 2.3. Capillary Electrophoresis

Capillary electrophoresis (CE) separations were conducted on a Beckman Coulter P/ACE MDQ capillary electrophoresis system equipped with 488 nm laser-induced fluorescence (LIF) detection. The capillary was rinsed using pressure with the separation buffer for two (2) minutes, and then the sample was injected (pressure) for 5 seconds. Separations were conducted at 15 kV for 15 minutes. After separation, the capillary was rinsed using pressure with pure water for five minutes. Capillary conditioning using 1 M NaOH was conducted with a 5-minute rinse as needed. Separation buffers tested included 10 mM carbonate (pH 10) and 10 mM carbonate with 12 mM SDS (pH 10).

### 2.4. Data Analysis and Figure Generation

Resulting electropherograms were generated and exported in comma-separated values format using 32 Karat software (Beckman Coulter Inc.). These files were imported into PeakFit (Systat) for smoothing (0.1% Loess) and baseline correction prior to peak fitting. The resulting smoothed and baseline corrected electropherograms or data from peak fitting was imported into Origin (OriginLabs) to generate figures. Chemical equations were drawn in ChemBioDraw Ultra. All raw figures were imported into Adobe Illustrator for image cleanup.

## 3. Results and Discussion

**Figure 1:**
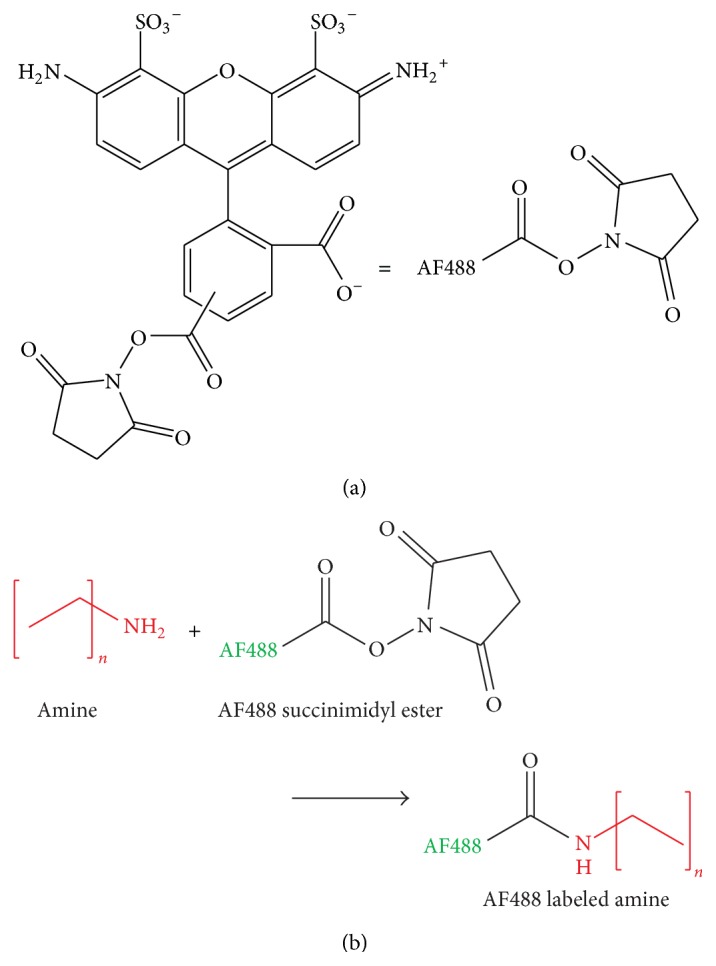
AlexaFluor 488 (AF488) succinimidyl ester and its reaction with primary amines. (a) The chemical structure of the reaction of AF488 succinimidyl ester with primary amines. N-hydroxysuccinimide acts as a leaving group to promote the formation of an amide bond to link AF488 to the primary amine group.

**Figure 2:**
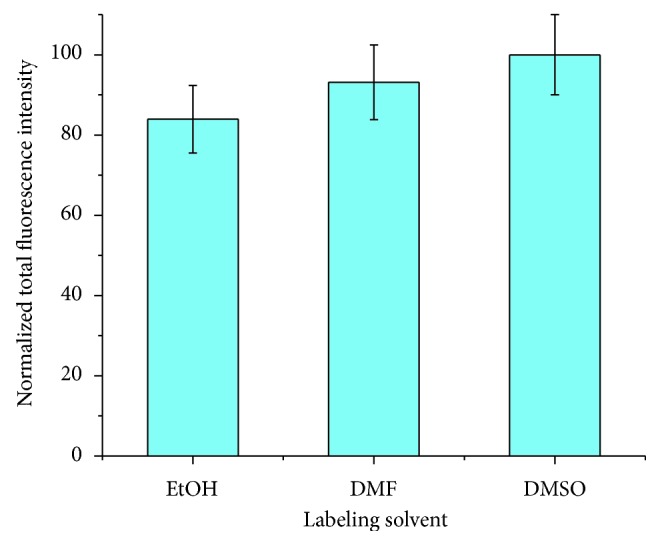
Normalized total fluorescence intensities of amines analyzed by CE-LIF and labeled with AF488 in ethanol (EtOH), dimethylformamide (DMF), and dimethylsulfoxide (DMSO). Labeling was conducted at 25 *μ*M amine, 50 *μ*M AF488 with 1 mM DIEA in the indicated solvent and incubated overnight. For analysis, the labeling solutions were diluted 1 : 20 in separation buffer (10 mM carbonate, 12 mM SDS, pH 10).

Figure 1 shows the labeling reaction between AF488 NHS-ester and a primary amine. This reaction is base-catalyzed and was found to proceed for C9-NH_2_ and shorter amines in 10 mM aqueous carbonate, pH 10. Longer-chain amines (C12-NH_2_ and longer) were found to be insoluble in aqueous solutions without surfactant and therefore did not label to any detectable extent. For this reason, we examined organic solvents for labeling reactions with DIEA included to provide a basic environment. The fluorescence intensities of amines labeled in 10 mM DIEA in ethanol, DMF, and DMSO and then separated in 10 mM carbonate, 12 mM SDS, pH 10, are shown to be normalized to the DMSO fluorescence intensity in Figure 2. Labeling proceeded to nearly the same extent, within error, in all three organic solvents. DMSO may provide slightly better labeling than ethanol. DMSO is often favored in extraction and sample preparation for its solvating ability and stability, so it is encouraging to see that labeling proceeded optimally in DMSO. However, these results also indicate that choice of solvent can be dictated by concerns such as downstream analysis method, safety, and ease of evaporation with marginal reduction in labeling.

**Figure 3:**
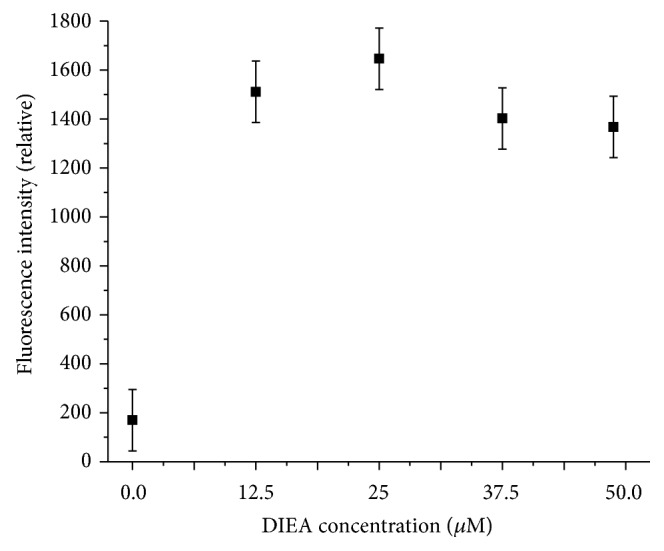
Effect of varying DIEA concentration on peak intensity (a measure of labeling efficiency). Relative fluorescence intensities of dodecylamine are plotted against varying DIEA concentration. Labeling was conducted at 1 *μ*M amine, 25 *μ*M AF488 with the indicated concentration of DIEA in ethanol and incubated overnight. For analysis, the labeling solutions were diluted 1 : 10 in separation buffer (10 mM carbonate, 12 mM SDS, pH 10). Error bars are calculated from triplicate labeling at 12.5 *μ*M.

To explore the impact of DIEA concentration on labeling efficiency, its concentration in ethanol was varied from 0 to 48.75 *μ*M in a solution that contained 1 *μ*M amine and 25 *μ*M AF488 NHS-ester.  Figure 3 shows the results of CE separation after an overnight incubation of the solutions. While very low levels of amine were labeled without any DIEA, there is no change, within error, of the amount labeled in solutions containing between 12.5 and 48.75 *μ*M DIEA. However, some contamination in the shorter chain amine region (C2-NH_2_, C3-NH_2_) was observed to increase with increasing DIEA concentration. These results indicate that DIEA concentration can and, when possible, should be kept to a minimum.

**Figure 4:**
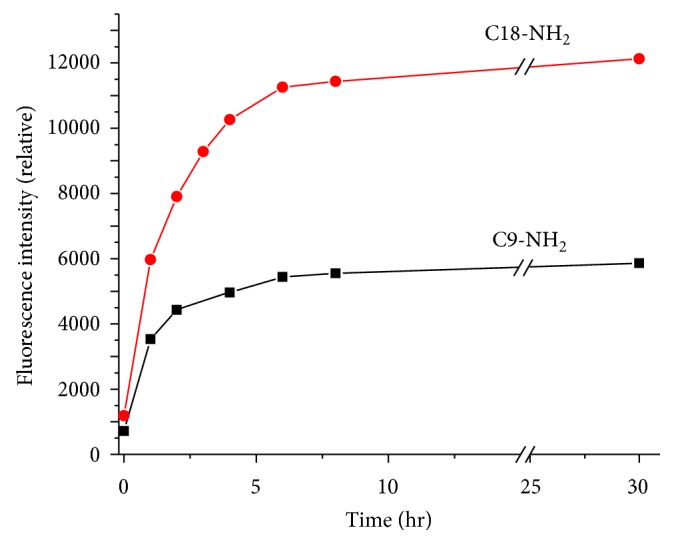
Effect of incubation time on labeling efficiency. Normalized fluorescence intensities of nonylamine and octadecylamine are plotted against incubation time. Labeling was conducted at 1 *μ*M amine, 25 *μ*M AF488 with 12.5 *μ*M DIEA in DMSO and incubated for the indicated time. For analysis, the labeling solutions were diluted 1 : 20 in separation buffer (10 mM carbonate, 12 mM SDS, pH 10).

While overnight incubations are logistically simple for an operator and for potential automated implementations, sometimes it is preferable to obtain results from a sample within a shorter window of overall time. Therefore, we examined the impact of incubation time on the labeling reaction extent.  Figure 4 shows the relative fluorescence intensity of the amine peaks diluted into separation buffer and immediately separated via CE at incubation times from 1 hr to 30 hr. Over 90% final intensity was achieved within 6 hours for the amines studied. While the samples were prepared in such a way to potentially yield pseudo-first-order kinetics, the data do not fit a rate law first-order in amine concentration and in fact most closely fit a rate law second-order in amine concentration. This does not seem like a physical likelihood given stoichiometry of the reaction and the standard mechanism proceeding via a tetrahedral intermediate. Therefore, we cannot make any statements about true kinetic parameters of the reaction based on our data and recommend a true kinetics study as part of future work with the AF488 NHS-ester fluorescent probe.

**Figure 5:**
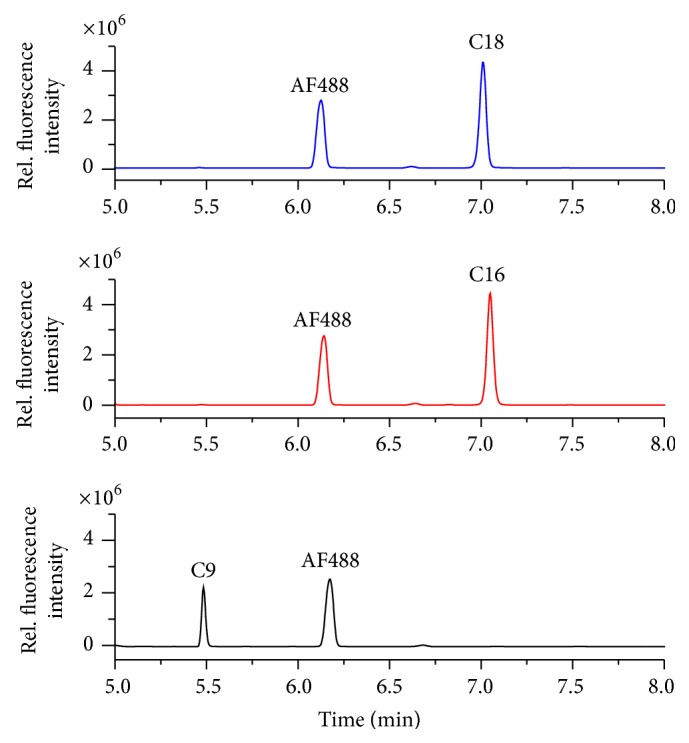
Electropherograms of amines labeled with the optimized labeling conditions (1 *μ*M amine, 25 *μ*M AF488 with 12.5 *μ*M DIEA in DMSO, and incubation for approximately 24 hours). For analysis, the labeling solutions were diluted 1 : 20 in separation buffer (10 mM carbonate, 12 mM SDS, pH 10).

Based on the above experiments, the optimum conditions for our work using AF488 NHS-ester as a fluorescent probe for amine analysis are 12.5 *μ*M DIEA in DMF with at least 6 hr and preferably overnight incubation. Electropherograms of amines separated in 10 mM carbonate, 12 mM SDS, pH 10, after labeling with the optimized conditions, are shown in Figure 5. While the separation conditions were not optimized nor studied in great detail in this work, separation characteristics are given in Table 1. Limits of detection (*S*/*N* = 3) were determined using the optimized labeling conditions and these separation conditions: 17 nM C16-NH_2_ and 5.7 nM C18-NH_2_. A contamination issue with our C9-NH_2_ stock prevented determination of its LOD during the timeframe of this work. This work provides a foundation for further work more fully optimizing an AF488-based amine assay for a desired application.

## 4. Conclusions

Amine detection using LIF with AF488 as the reactive probe enables potentially novel analytical techniques as it provides an alternative to other probes' fluorescent at 488 nm. The optimized reaction conditions are mild and fast (90% efficiency within 6 hrs) while enabling excellent limits of detection (nM or ppb). While AF488 is not fluorogenic and therefore cannot be used as a fluorescent probe without some form of postreaction separation, we have demonstrated that this separation can be achieved using a simple, standard MEKC solution and a commercial CE instrument. The limits of detection achievable using the commercial system and initial CE separation conditions were in the low nM range, or single parts-per-billion, making this technique more than sufficiently sensitive for multiple applications. Further work remains to fully characterize the kinetics of the labeling reaction. Additionally, the current separation method is insufficient for the simultaneous analysis of C16-NH_2_ and C18-NH_2_; thus, further work is required to develop application-specific optimized separation methods.

## Figures and Tables

**Table 1:** Table of separation characteristics.

Amine	Amplitude	Area	Elution *T* ^a^ (min)	Peak efficiency (Theor. plates)	Peak effici. (plates/m)	LOD (nM)
Nonylamine	2.2 × 10^7^	3.5 × 10^7^	5.5	2.7 × 10^5^	550000	N.D.^b^
Hexadecylamine	4.5 × 10^7^	1.1 × 10^8^	7.0	1.7 × 10^5^	350000	17
Octadecylamine	4.4 × 10^7^	1.1 × 10^8^	7.0	1.1 × 10^5^	220000	5.7

^a^Elution time.

^b^N.D.: not determined.
